# Control and Prevention of Epizootic Lymphangitis in Mules: An Integrated Community-Based Intervention, Bahir Dar, Ethiopia

**DOI:** 10.3389/fvets.2021.648267

**Published:** 2021-11-12

**Authors:** Bojia E. Duguma, Tewodros Tesfaye, Asmamaw Kassaye, Anteneh Kassa, Stephen J. Blakeway

**Affiliations:** ^1^The Donkey Sanctuary-Ethiopia Office, Addis Ababa, Ethiopia; ^2^Director, International Department, The Donkey Sanctuary, Sidmouth, United Kingdom

**Keywords:** epizootic lymphangitis, Bahir Dar Ethiopia, EZL prevention and control, mule, community-based animal health care

## Abstract

From 2010 to 2017, as part of a wider animal welfare program, The Donkey Sanctuary piloted an integrated, community-based model for the control and prevention of epizootic lymphangitis (EZL) in cart mules in Bahir Dar, Ethiopia. Stakeholders included muleteers, service providers, and transport and animal health regulatory authorities. Interventions included muleteer education, wound prevention, harness improvement, animal health professional training, treatment of early EZL cases, euthanasia for advanced cases, and review of transport services and traffic guidelines. The project followed a participatory project management cycle and used participatory learning and action tools to facilitate stakeholder engagement and ownership. Participatory and classical epidemiology tools were employed to raise and align stakeholder understanding about EZL for effective control and prevention and to evaluate the progress impact of the model through annual prevalence surveys. During the intervention, the annual prevalence of EZL reduced from 23.9% (102/430) (95%CI: 19.8%−27.0%) in 2010 to 5.9% (58/981) (95% CI: 4.4%−7.4%) in 2017, and wound prevalence from 44.3% in 2011 to 22.2% in 2017; trends in the reduction of the prevalence maintained in the face of a mule population that increased from 430 in 2010 to ~1,500 in 2017. While non-governmental organization (NGO)-led interventions can facilitate change by trialing new approaches and accessing new skills and resources, sustainable change requires community ownership and strengthening of service provision systems. To this effect, the project raised muleteer competence in mule husbandry and EZL prevention strategies; strengthened veterinary competence; facilitated more mule-friendly traffic, transport, and waste disposal guidelines and practices; supported mule-community bylaws to control EZL; and established a supportive network between stakeholders including trusting relationships between muleteers and veterinary services. To advance the intervention model in other endemic areas, we recommend elucidation of local epidemiological factors with other stakeholders prior to the intervention, early engagement with veterinary and transport service regulatory authorities, early development of bylaws, exploration of compensation or insurance mechanisms to support euthanasia of advanced cases, and additional social, economic, and epidemiological investigations. In line with the OIE Working Equid Welfare Standards, we suggest that integrated community-based interventions are useful approaches to the control and prevention of infectious diseases.

## Introduction

This article reports on a working mule welfare project (the project) that ran from 2010 to 2017 and focused on the control and prevention of wounds and the disease epizootic lymphangitis (EZL) in Bahir Dar, Amhara Region, as an indicator of the success of a more extensive participatory, community-based donkey and mule welfare program in Ethiopia facilitated by The Donkey Sanctuary (TDS), a United Kingdom-based donkey and mule welfare charity.

Around 13.3 million working equids provide essential services in Ethiopia (1). Their contribution to the economy remains generally under-recognized, and services available to the sector are therefore generally poor (2–4).

Around 380,000 of these working equids are mules, with ~140,000 living in the Amhara Region where ~56,000 power carts (1). The Amhara Region also has one of Ethiopia's largest populations of working donkeys. Mule numbers in Bahir Dar, capital of the Amhara Region, are increasing, from 430 in 2010 to around 1,500 in 2017, alongside increasing development and a human population in Ethiopia that doubled between 1995 and 2019 (5).

All mules in Bahir Dar power carts. They transport building materials (timber, stone, and cement) and agricultural goods (seeds, fertilizers, pesticides, and harvested produce) to and from markets, grain and flour to and from grinding mills, water where there is no piped supply, solid waste to municipal dumps, and people (6, 7).

Mules represent a significant investment, make a substantial (sometimes the only) financial contribution to muleteer household economies (6, 8), and provide valuable low-carbon community services, thereby serving the Sustainable Development Goals (9). Yet lack of knowledge; poor husbandry; harness wounds; lameness; colic; infectious diseases such as EZL, African horse sickness, tetanus, strangles, and parasites; and a lack of relevant support services all compromise welfare and productivity (7, 8, 10–14). On average, a mule affected with EZL in Gondar, Amhara, is estimated to cost its owner around ETB 6,000 per year (~GBP 100, July 2021) in lost production (15).

EZL is the most visible and prevalent of the infectious diseases affecting equids in Ethiopia (16–21). In endemic areas, emaciated, abandoned horses and mules with the running sores of advanced EZL can be seen standing in the middle of busy roads where the breezes from passing vehicles provide some respite from flies. Welfare assessment, using the Hand (22–25), identified mule–muleteer–societal relationships, wounds, and EZL as the main welfare challenges to be addressed in this project. Lameness and nutrition would also be addressed tangentially.

EZL is primarily a chronic contagious disease of equids (17, 18, 20, 26), with horses being most susceptible, donkeys least susceptible, and mules in between. The causal agent is *Histoplasma capsulatum* var. *farciminosum* (HCF), a dimorphic fungus (one that can exist in both unicellular (yeast) and filamentous (mycelial or mold) states) which can live independently in soil, making eradication difficult. It is endemic to Ethiopia (27), particularly in the hot, humid upland areas between 1,500 and 2,800 m above sea level (20).

Control of EZL is challenging, with no completely satisfactory treatment (26). Spread between equids is thought to be facilitated by the presence of open wounds, close contact, flies, and poor work, hygiene, or husbandry practices (28). Treatment requires continuing owner compliance. Early identification and intensive follow-up are critical for successful therapy (17, 29). The more advanced the disease, the more guarded the outcome (30, 31). Tincture of iodine (2%) can be used topically, and sodium or potassium iodide can be parenterally administered via drinking water or feed, although lengthy treatment can lead to iodine toxicity. All are available in Ethiopia. The antifungal drug amphotericin B is generally impractical for working equids because of the specialized treatment protocols, potential side effects, and requirement for close monitoring (32). There is no readily available commercial vaccine for prevention, although an attenuated vaccine and a killed formalized vaccine are reported to have been used for its control in some endemic areas of west Asia (27, 33). Currently, therefore, the only viable means of prevention and control of EZL involves close collaboration between mule-using communities, veterinary services, and regulatory authorities, with cases caught early and intractable cases humanely euthanized.

The TDS program in the Amhara Region followed a strategic review with a move from direct veterinary intervention services to community-based approaches measured against welfare outcomes that could better deal with the technical, social, and economic complexities of donkey and mule welfare including the multifactorial nature of a disease such as EZL (34). It drew on lessons from community-based animal healthcare (35, 36) and participatory epidemiology (37, 38) including the global eradication of rinderpest in cattle (39–41).

In summary, the project trialed a community-based approach to understanding and improving cart mule welfare, in a location where wounds and EZL were the most visible welfare challenges with the explicit intention of exploring sustainability.

## Methods

### Materials

#### Project Study Area

The study was conducted in cart-pulling mules of Bahir Dar city, located next to Lake Tana, source of the Blue Nile, in the Amhara Region, northwestern Ethiopia, at an altitude of 1,820 m above sea level. The average annual rainfall is 1,416 mm. The short rainy season is in March and April, and the long rainy season is from June to September. Bahir Dar has a borderline tropical savannah climate with an average low temperature of 11.7°C and an average high temperature of 26.7°C. The mean relative humidity of Bahir Dar is 58.4% (42).

#### Project Study Population

The Bahir Dar City Administration provided a mule population estimate of around 500 mules for 2010 at the start of the project. The mule cart sector was informal, mules were not registered nor licensed, and there was little formal engagement between the municipality and sector, so the estimate was based on little data. The baseline survey which aimed for a full census found 430 mules. This number had risen to nearly 1,500 by the end of the project. During the project, the municipality based its annual mule population estimates on project census surveys, rather than its usual general annual estimate of animals in the city. Official full census surveys are conducted every 10 years in Ethiopia. Annual mule numbers are presented in Table 1, and the survey methodology is explained in Section Annual cross-sectional surveys: prevalence of EZL and wounds.

**Table 1:** Number of mules sampled for annual cross-sectional survey of EZL and wounds, 2010–2017 (baseline and implementation surveys).

**Survey year**	**Number of mules sampled**	**Survey method**
2010*^a^*	430	Census survey
2011	623	Census survey
2012	1,128	Census survey
2013	1,266	Census survey
2014	NA*^b^*	No survey conducted
2015	394	Sample survey
2016	1,436	Census survey
2017	981	Sample survey

a *The project baseline survey*.

b *NA—not applicable*.

Cart mule demographics were fluid. Almost every mule had a dedicated handler (muleteer) who chased work across the city independently, and there appeared to be no stable groupings of mules. Owners with multiple mules generally rent them out in long-term arrangements with the muleteer responsible for almost all aspects of husbandry; and cart mule associations appeared to be loose affiliations. As mule population increased alongside diversification of work opportunities, competition also increased with larger numbers of mules accumulating at collection sites.

Nevertheless, the muleteer community was self-aware. Apart from business risks, EZL was its greatest threat, and many muleteers were aware of the disease and its status across the population. Muleteers were initially wary of the project, but once they understood the project's approach and intentions, most became more trusting. Mules are only kept to work, and working mules are visible, so key informants from within the muleteer community played an essential role in the project's success, helping project staff to find muleteers with mules affected by EZL including in the outer reaches of the city, both during the intervention and the annual surveys.

### Project Study Framework

TDS identified the cart mules of Bahir Dar as a target project for two strategic reasons. First, the welfare of cart mules with wounds and EZL is visibly compromised. Second, the visibility of these mules in a regional capital helped raise awareness about working animals more generally and so fed into the awareness-raising and advocacy strand of TDS's broader strategy.

**Figure 1:**
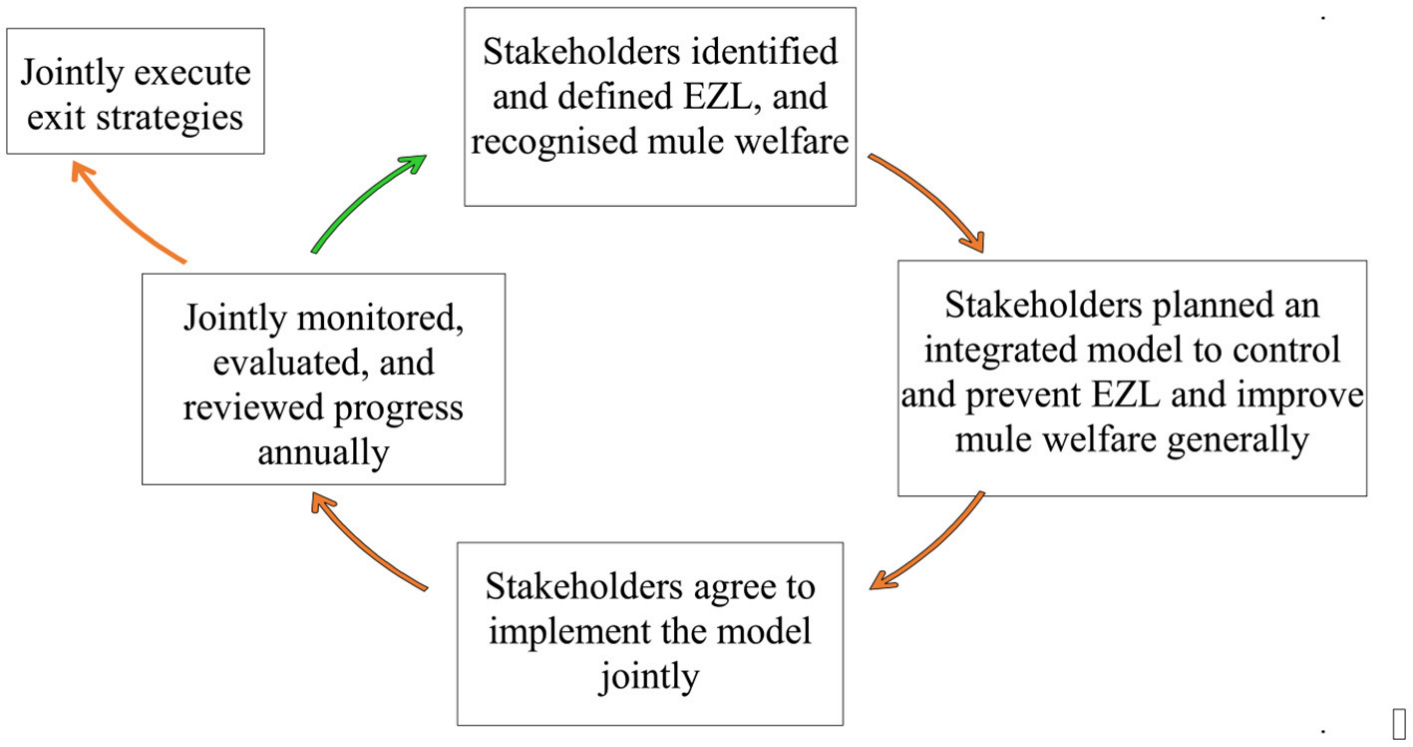
EZL participatory project management cycle (PPMC). Review has been a continuing process within PPMC before implementing the exit strategies. Exit strategies can happen at different times for different components of the project.

The project followed a participatory project management cycle (PPMC) which allows flexibility, accumulation of learning through review points, and the ability to amend plans and activities in agreement with other stakeholders (43, 44). The cycle can be described as stakeholders identifying and defining the problem together, then planning together, then implementing together, and then monitoring and reviewing together, before starting the cycle again by planning the next stage together, taking into consideration lessons learnt in the previous cycles. Awareness of the need for an exit strategy is explicit from the start, so additional cycles aim increasingly to hand over aspects of the work, while focusing on specific areas of challenge. Also explicit is the need to reduce external contributions to the project over time. This is shown schematically in Figure 1.

While TDS identified EZL in mules in Bahir Dar as a social, economic, and animal health and welfare problem, it then needed to involve other stakeholders for the project to work.

### Stakeholder Analysis

Stakeholder analysis started by talking with obvious stakeholders such as mule owners and users (muleteers), veterinary service providers, and regulatory officials. TDS then used interviews and stakeholder analysis (45) with these individuals and emerging key informants to understand the wider range of stakeholders, their areas of interest and influence, and the roles they might play in making the project a success.

### Epidemiology

The application of epidemiology and epidemiological tools served two main roles during this EZL control and prevention project: to engage all stakeholders with an agreed understanding about EZL prevention and control based on both local knowledge and published articles; and to provide a reliable methodology for annual EZL prevalence surveys for project monitoring and evaluation.

#### Case Definition and Diagnosis of EZL

The case definition for EZL in this study was a working mule in Bahir Dar with a diagnosis of EZL based on clinical examination with confirmation through direct microscopy.

The clinical examination protocol for EZL involved inspection of the entire skin surface for ulcerative wounds or suppurative spreading dermatitis and lymphangitis and palpation along the lymphatic vessels and lymph nodes for the presence of nodules. Cases were characterized as advanced by the presence of button-type ulcers or nodules, cording or thickening of lymphatic vessels, or functional abnormalities such as lameness, dyspnea, or visual impairment.

Swab samples from any suspect lesions, including ulcerating harness-related lesions, bit sores, or ocular discharge, and aspirates of nodules along the lymphatic vessels or lymph nodes were collected for direct microscopy. Direct microscopic examination of samples was conducted in collaboration with the Bahir Dar Regional Veterinary Laboratory. Both Gram's and Giemsa stains were applied to examine for pleomorphic yeast-like cells with a halo appearance under the oil immersions lens (29).

#### Annual Cross-Sectional Surveys: Prevalence of EZL and Wounds

A baseline survey to assess general welfare, including prevalence of EZL and wounds, was conducted by the TDS staff in 2010 before making a final commitment to implement the project. This was a full census survey aiming to reach every mule and involved clinical examination of mules for EZL, open wounds, harness fit and quality, lameness, sex, age [estimate based on dentition (46)], and body condition score [BCS, using a five-level scoring methodology (47)] as potential risk factors for EZL. General observations were also made regarding commodities being transported, working practices, and mule–muleteer relationships. The project monitoring and evaluation strategy was to resurvey mules each year to assess the effectiveness of its protocol by measuring annual change in EZL and wound prevalence. In view of the growth and demographic complexity of the mule sector and because the project was about general welfare as well as EZL, the preferred strategy was to continue with full census surveys, to reach all mules, sample each mule for EZL, and check for open wounds, collect accurate population data, and check general welfare across the growing population of working mules. Resampling of mules was avoided by registering muleteer names during the exercise and marking each mule on the forehead with permanent red ink.

Surveys were conducted by a team composed of veterinarians from Bahir Dar Clinic and Bahir Dar Regional Veterinary Laboratory and TDS staff. To meet the primary objective of controlling EZL, every effort was made to ensure inclusion of each and every mule working, at rest, grazing, left at home, or abandoned for the success and progress impact of the intervention model. Muleteers increasingly assisted in the process as they became more aware that every case is a potential source of infection and needs to be reported as soon as discovered. Other key informants, including the Kebele administration, were also involved in the organization of the census survey. Surveys took place during the month of May when EZL cases are higher and were completed over 2 to 3 weeks.

For reasons outside project control, there was no survey in 2014, and full census surveys were not possible in 2015 and 2017. In 2015, the sample size was calculated based on the 21% prevalence published in a previous Ethiopian study of EZL prevalence in cart mules (17). Assuming 21% prevalence with 95% confidence level, 80% power of test, 5% precision and an estimated population size of 1,400 mules required a minimum sample number of 380 (48), and the project sampled 394 mules. In 2017, because prevalence had risen in 2016, the project reduced the margin of error, assumed prevalence of 50%, precision level of 3% and, with an estimated population of 1,500 mules, calculated a minimum sample number of 889, and 981 mules were sampled (49). Mule selection in 2015 and 2017 used systematic random sampling where every other mule was sampled, and planning together with key informants from the mule community ensured sampling of mules across the full geographic and demographic range in and around Bahir Dar.

#### Stakeholder Contributions and Ownership of the Interventions

For project success, all stakeholders needed to reach a common understanding about EZL and what they could do to minimize or prevent it.

With the use of two participatory epidemiology approaches, key informant interviews, and focused group discussions (FGDs), factors associated with EZL were explored using a range of participatory exercises. The purpose of these exercises was to build engagement; to respect and understand local perceptions; and to check that these aligned generally with published epidemiology on EZL. These exercises aimed to allow stakeholders, whatever their level of education or literacy, to contribute their knowledge and experience, share in the analysis, and take ownership of the prevention and control strategy.

The key informant interviews and FGDs were conducted primarily by the TDS program staff. Themes and results were shared with the wider community in subsequent meetings and training sessions through narrative, reporting of ranking results, and map building (37, 50, 51).

##### Key Informant Interviews

Individual, semi-structured, face-to-face interviews were conducted with 12 key informants. These included five experienced muleteers, one municipality officer, two animal health professionals, two traffic police officers, one senior livestock officer, and one manager of a private waste collection business. Each interview involved a set of questions to open wider conversations that allowed interviewees to add ideas that might not have occurred to the interviewer.

Questions explored the socioeconomic impact of EZL on cart mules in Bahir Dar; factors predisposing to EZL; challenges to controlling and preventing EZL; the roles of the different institutions and associations; and what should be done about EZL.

##### FGDs

Five FGDs were held to explore muleteer perceptions about EZL and its epidemiology. Participation criteria were agreed with stakeholders such that all participants were experienced muleteers, were members of cart mule associations, and had some form of social responsibility in the community as well as passion and willingness to participate. Muleteers new to the carting business in Bahir Dar who lacked background about EZL and its impact on the city and children driving carts for their older relatives were therefore excluded.

Around 30 mule-related associations were registered with the Bahir Dar municipality, but some were short term, for example, as part of a small microfinance scheme, and were no longer functional. Participants in the FGDs came from the eight most active associations based on the large size of membership, longer years of establishment, integrity of their bylaws, and diverse work type and geographic locations. Despite being members of an association, muleteers work independently, and FGDs were mixed depending on when different muleteers had the time to join a session. Location and timing of FGDs were dictated by the muleteers and their daily schedules.

Topics were explored in the FGDs with the help of participatory learning and action (PLA) tools (50, 51), which allow participants to represent their worlds, their knowledge, and their experience, in a variety of ways including diagrams, pictures, and maps. The discussions between participants as they create the representations, the representations themselves, and the detailed discussion afterwards are all equally important parts of the process. PLA tools help ensure that all participants contribute, even the quietest ones. When facilitated well, the process holds attention beyond that of a general discussion, allowing every point to be fully examined.

The topics explored (and the PLA tools used) were as follows:

- *Socioeconomic impacts of EZL* (brainstorming and discussion);
- *Predisposing factors to EZL*: identify (brainstorming) and rank and discuss (simple ranking);
- *Pairwise ranking*: using the top risk factors identified in the previous exercise, compare and rank these risk factors in pairs;
- *Seasonal calendars*: draw a line that represents your working year; mark the four main seasons—Tseday, Bega, Belg, and Kiremt; add in other major events in the year, e.g., types of work that may vary during the year, and times of the year when EZL cases are lowest and highest; and explain and discuss; and
- *Mapping Bahir Dar from a mule perspective*: participants worked on a map of the city showing collection sites including feeding and watering points, parking, markets, construction sites, and grain mills, adding other meeting sites of importance and marking routes used to move through the city while discussing the challenges they faced (participatory mapping).

### Planning and Implementation

Once the various stakeholders had established a common understanding about the problem, TDS facilitated a series of consultative workshops to agree on a collective implementation plan.

Workshops started with a summary of project progress to date (as in Sections Project study framework–Epidemiology) and then used a problem tree exercise (52) to explore root causes and intervention points.

These meetings helped identify key intervention activities, reach an agreement on how the work should be monitored and evaluated, assign roles and responsibilities, and reach an agreement on other implementation modalities and a timeline. The idea of an exit strategy for TDS involvement was introduced from the start to provide focus.

Facilitated learning including practical training—for all stakeholders (including TDS) but most actively for muleteers, harness makers, and animal health professionals—was a central theme, as was ensuring availability of treatment for treatable cases and euthanasia for incurable cases. Other activities aimed at changing attitudes, behaviors, and practice regarding mule transport within the municipality transport officials and traffic police.

The project started with an official public launch. Stakeholders needed to commit to being part of the “project team” before the official launch to help build trust, to reinforce that the project could only succeed as a joint effort, and to mitigate against groups dropping out.

#### Education of Muleteers

Muleteer education was practical and participatory. It targeted all muleteers and responded to points raised by all stakeholders including the muleteers themselves. TDS was involved with the local veterinary staff in improving muleteer understanding and practical knowledge of mule behavior and handling; improving general mule care and management; and reducing wounds and improving hygiene and segregation practices around EZL. Transport officials and traffic police worked together with muleteers on traffic-related education.

Specifics included herd health and EZL prevention (where herd can mean all the mules in Bahir Dar or a subset, such as those belonging to one muleteer, or all members of a cart mule association, if they can be managed in any way separate from other mules); use of improved harness (saddle, bits, and straps made of natural materials) and implications of sharing harness; daily mule checks for early identification of all health problems including wounds and EZL; early treatment including how to engage with veterinary services; principles and practice of wound management (including application of tincture of iodine); case segregation while housing, feeding, and being at collection sites; understanding the concept of euthanasia and how it supports control and prevention of EZL; and safe road use including knowledge of road safety regulations and good communication while driving.

Mule welfare training modules were initially developed and delivered monthly by TDS staff and then through training delegates selected by their cart mule associations who provided training to other muleteers, including new muleteers outside their own association. The treatment protocol for active EZL cases required muleteers to clean wounds and apply tincture of iodine initially under the direct supervision of an animal health professional. This training support was later taken over by local animal health professionals. Harness training was facilitated by TDS harness specialists (animal health assistants trained by TDS international harness specialists). Road safety training was facilitated by local traffic police officers.

#### Training of Harness Makers

Almost all cart mule wounds were related to poor harnessing practice, and the project therefore needed to address this. It developed, tested, and piloted improved saddle prototypes, humane bits, and canvas straps. Training was provided to 12 harness makers stationed at two locations in Bahir Dar on the making, fitting, repair, and maintenance of this harness. TDS initially supplied the raw materials, e.g., wood, canvas, and the bit-making tools, during the prototype and testing phase.

#### Training of Animal Health Professionals

Local animal health professional staff included veterinary surgeons (6-year university training), BVScs (Bachelors of Veterinary Science, 3-year university training), and animal health assistants (3-year technical college training) at the public veterinary clinics in central Bahir Dar, Meshanti, Zanzelima, Gonbat, and Tis-Abay. Except central Bahir Dar with six to eight veterinary staff, other public veterinary clinics were health posts with one to two veterinary staff. There were also around seven or eight private animal health practitioners across Bahir Dar. Turnover among veterinary staff in public clinics, particularly the small health posts, was high, although some interested individuals remained engaged with the project even after promotion.

Before the project, muleteers were not taking mules with EZL to veterinary clinics for two reasons: because the animal health staff lacked competence in EZL diagnostics, treatment, and euthanasia (and equid medicine generally) and because clinics lacked the necessary drugs.

TDS therefore trained the animal health professionals and equipped the public veterinary clinics with tincture of iodine (2%) and potassium iodide.

Animal health professional training was prepared in four modules: mule behavior and handling; EZL diagnosis, treatment, and euthanasia; EZL epidemiology and herd health; and community facilitation skills and equine husbandry. Each module was delivered as a Continuing Professional Development (CPD) course and followed up with more informal practical hands-on training in the field at veterinary clinics.

Once the project was established, veterinarians with competence to train others (see Section Monitoring and evaluation of the project), including in communication skills, were selected for Training of Trainer (ToT) training so that they could continue to train muleteers and deliver further CPD training to other animal health professionals as necessary. Trainees were followed monthly by the TDS team up until 2015 and semiannually thereafter. The project targeted all animal health professionals for training.

#### Treatment of Early EZL Cases and Euthanasia of Advanced Cases

Treatment and euthanasia were critical elements of the intervention to contain spread of EZL because untreated or abandoned EZL mule cases remain a source of infection for other mules in Bahir Dar.

Cases were classified as early, established, advanced, and untreatable (recommended for euthanasia). Treatable cases were treated by incision of nodules when present, application of tincture of iodine, and administration of parenteral iodides (potassium iodide, Ubiche) in drinking water (30). Length of treatment increased the more severe the case classification. For the detailed procedure and outcome, please refer to Supplementary Section of the manuscript.

Euthanasia was performed using intravenous injection of barbiturates (Pentoject® 200 mg/ml solution, pentobarbital sodium 20% w/v, XVD132, Animalcare Limited, UK).

### Monitoring and Evaluation of the Project

Progress indicators for each aspect of the project were identified and agreed on by all stakeholders. Annually repeated cross-sectional surveys were conducted to assess prevalence of EZL and wounds as key indicators for the success of the project (see Sections Project study population and Annual cross-sectional surveys: prevalence of EZL and wounds).

Training was assessed using the TDS four-level general competence framework for trainees: starter, becoming independent, independent, and trainer (see Table 2).

**Table 2:** The Donkey Sanctuary general competence framework for trainees.

**Competence level**	**Description and assessment process**
Starter	All trainees start at this level to acknowledge and encourage interest. No real knowledge or experience but with active interest.
Becoming independent	Acknowledgement of starting actively along training journey. An active trainee, a reflective learner, with good attitude and regular attendance.
Independent	A trainee who has continued to show competence in what was taught and to demonstrate reflective practice, for at least 6 months after completing the formal training. Assessed by the TDS staff through practical follow-up field work.
Trainer	Independent practitioner in a primary area of competence, with additional independent competence following Training of Trainers (ToT) training. Identified for ToT training by demonstrating good communication skills, an interest in helping others to learn, strong reflective practice, and an interest and enthusiasm for becoming a trainer, and who has then reached independent level in training practice (assessed by the same competence framework).

Field reports were prepared monthly, project progress reports were prepared quarterly, and project evaluation reports were prepared annually. All stakeholders took part in consultative review workshops. External evaluations were conducted by Amhara Region regulatory signatories (Bureau of Livestock Agency and Bureau of Finance and Economics) midterm and at the end of the 5-year project agreement between TDS and the Government.

### Project Exit Strategy

The project started with a 5-year agreement between TDS and the regional bureaus. The project aimed to find an approach to improving mule welfare, including reducing prevalence of EZL, that could be owned and sustained locally with a minimum of external input. Reflective learning among all stakeholder groups, including TDS, would be a central part of the work. TDS envisaged gradually reducing its involvement over further project cycles, while continuing to help refine the approach and institutionalize key components such as cart mule business, training, and animal welfare standards.

This would involve empowering communities, mainstreaming best practices into the relevant sectors, recognizing EZL as a notifiable disease by the Bahir Dar municipality, transforming the local veterinary clinics to handle EZL cases and euthanize advanced cases by the regional livestock agency, formalizing and regulating mule-powered transport by the transport sector, supplying improved harness by local harness makers, formulating local community bylaws among the cart muleteers, and ideally encouraging institutions and organizations such as veterinary schools, the Ethiopian Veterinary Association, or the Ethiopian Animal Health Assistant Association to review their curricula, strengthen communication and community-engagement practices, and offer equine CPD training.

Gradually refining and reducing involvement over time was the project's exit strategy—a tailing out rather than an abrupt end. However, this was not possible for reasons outside project control.

### Statistical Analysis

Information obtained from key informants was captured using facilitator notes and summarized into thematic areas and presented as a narrative. Results of FGDs obtained using different tools were presented in a map and a table.

Statistical analysis for annual prevalence study was carried out with STATA software version 11, using the chi-square statistical test, with the significance test set at a *p*-value < 0.05. Mule demographics and prevalence studies were presented using proportions.

To examine the statistical significance for the persistence of the prevalence reduction, one-way ANOVA trend analysis was executed for prevalence reduction of both EZL and wounds across the years.

## Results

### Demography—Survey of Mules and Muleteers

Owners sourced mules from local livestock markets around Bahir Dar including Yigodi, Merawi, and Bahir Dar central market. These mules are likely to have come through more distant markets in Este, Debre Tabor, and Adet, which are part of an equine trade network originating from South Wollo that extends across Central, Southern, and Western Ethiopia.

Mule transport is currently considered an informal economic activity and is unlicensed. Training and support services are limited, with no training available in mule handling, husbandry, or business management.

The major items transported by cart mules in Bahir Dar included construction supplies (56.6%) such as wood, stone, cement, gravel, and sacks of sand to meet the demand for Bahir City expansion; agricultural produce from nearby farms to markets (27.3%); and other commodities for sale and household use (16.1%) including water and flour.

Table 3 presents the age structure of the mule population of Bahir Dar in 2010 (extracted from 2010 baseline survey data), showing that 52% of mules were male and 48% of mules were female.

**Table 3:** Age structure of working mules in Bahir Dar in 2010 (number (N) and %), from baseline data.

**Mules < 5 years*N* (%)**	**Mules 5–10 years *N* (%)**	**Mules > 10 years*N* (%)**
47 (11)	241 (56)	142 (33)

As part of the baseline, the TDS team assessed the working condition of mules to be generally poor. Almost all carts and harnesses and harnessing systems were poor. All drivers use a stick or whip to drive mules. Mules work throughout the day; there was no shifting practice unlike cart horses in other areas of Ethiopia. They stay loaded without feed and water at collection sites for an extended period particularly on non-market days. Cart owners work with an EZL case until the disease advances to affect the mule's ability to work, for example, by its impact on locomotion or its respiratory system, and finally, they abandon the mule.

### Study Framework: Results of the PPMC Approach

Results of the PPMC approach with its collaborative working and flexible review points showed themselves in various ways.

Muleteers at the start mistrusted TDS and the veterinary services, used traditional treatments, and avoided outside interference. However, when they understood that the project was genuine about working with them and saw the potential benefits, they engaged with the veterinary service, with muleteers starting to accept euthanasia. Stakeholders took their own initiatives within the project structure: some cart mule associations introduced EZL bylaws; transport officers and traffic police in consultation with the cart mule associations developed mule-friendly improvements in traffic regulations supported with information bulletins put out through municipality media channels; and traffic police reported reduced numbers of road traffic accidents involving cart mules as the project progressed.

### Stakeholder Analysis

Active involvement of diverse stakeholders is a central aspect of a community-based approach. The full list of stakeholders engaged with this project are listed in Box 1, grouped by affiliation.

Box 1 **Project stakeholders by affiliation.**

**Project stakeholders**

**Muleteers**

Muleteers in general

Delegates from eight of the most active of the 30 cart mule associations

**Private sector**

Cart and harness makers

Private animal health professionals/clinics

Licensed solid waste management cooperatives

**Bahir Dar city administration**

Animal health professionals from Bahir Dar clinics and subclinics

Bahir Dar city municipality

Transport officers (make regulations)

Traffic police (enforce regulations)

Solid waste management staff

**Amhara regional bureaus**

Livestock Agency

Regional Livestock Officer

Lab technicians from Bahir Dar Regional Laboratory

Bureau of Transport

Bureau of Finance and Economics

Regional NGO desk

**The Donkey Sanctuary employees**

Animal health professionals: veterinarians and allied professionals including harness specialists

Social science staff

Education specialists

### Epidemiology

#### Case Definition and Diagnosis of EZL

**Figure 2:**
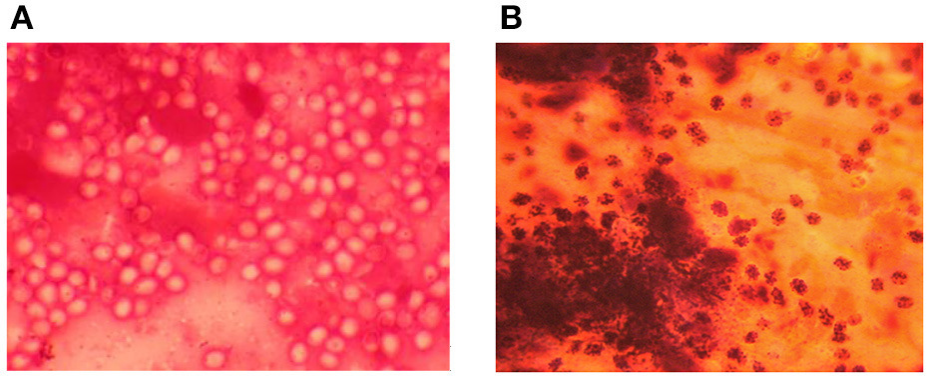
Photomicrographs of *Histoplasma capsulatum* var. *farciminosum* yeast cells showing intact nonstaining yeast cells sampled from an early case **(A)** and disintegrated yeast cells with deep-staining dotted granules from an advanced case **(B)**.

The case definition for EZL provided a common understanding of the disease for diagnosis and training. It was observed consistently during the study that intact and non-staining yeast cells of HCF were common in early cases of EZL, and disintegrated yeast cells with deep-staining dotted granules were more common in relatively advanced cases (see Figures 2A,B). These observations have not been reported in other studies and are therefore of note.

The case definition provided the basis for the annual prevalence surveys. The results from the prevalence survey are presented in Table 4.

**Table 4:** Prevalence of EZL in cart mules in Bahir Dar from baseline and annual prevalence surveys.

**Year**	**Test positive**	**Prevalence (%)**	**95% CI (%)**	**Total tested sample**
2010	102	23.9%	19.8%−28.0%	430
Total (baseline)	102			430
2011	79	12.7%	10.0%−15.3%	622
2012	54	4.8%	3.5%−6.0%	1,128
2013	75	5.9%	4.6%−7.2%	1,266
2014	NA*^a^*	NA	NA	NA
2015	23	5.8%	3.5%−8.2%	394
2016	144	10.0%	8.5%−11.6%	1,436
2017	58	5.9%	4.4%−7.4%	981
Total (project)	433			5,827

a *NA—Data not available*.

#### Epidemiology: Annual Change in Prevalence of EZL in Bahir Dar

**Figure 3:**
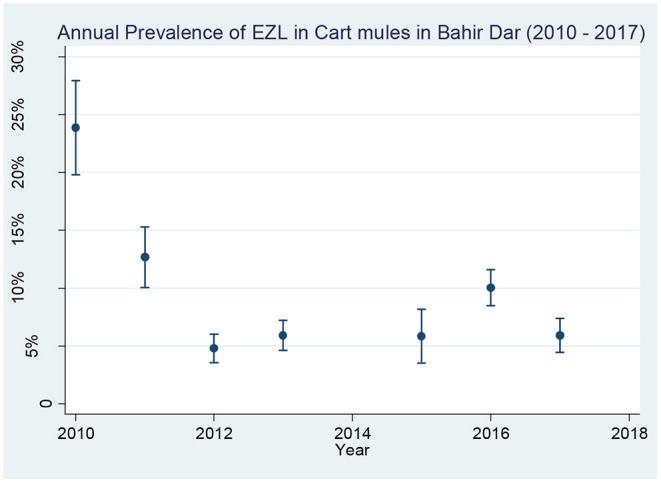
Annual prevalence of EZL in cart mules in Bahir Dar from 2010 to 2017 [NB: Error bars represent 95% confidence interval around EZL prevalence value].

**Figure 4:**
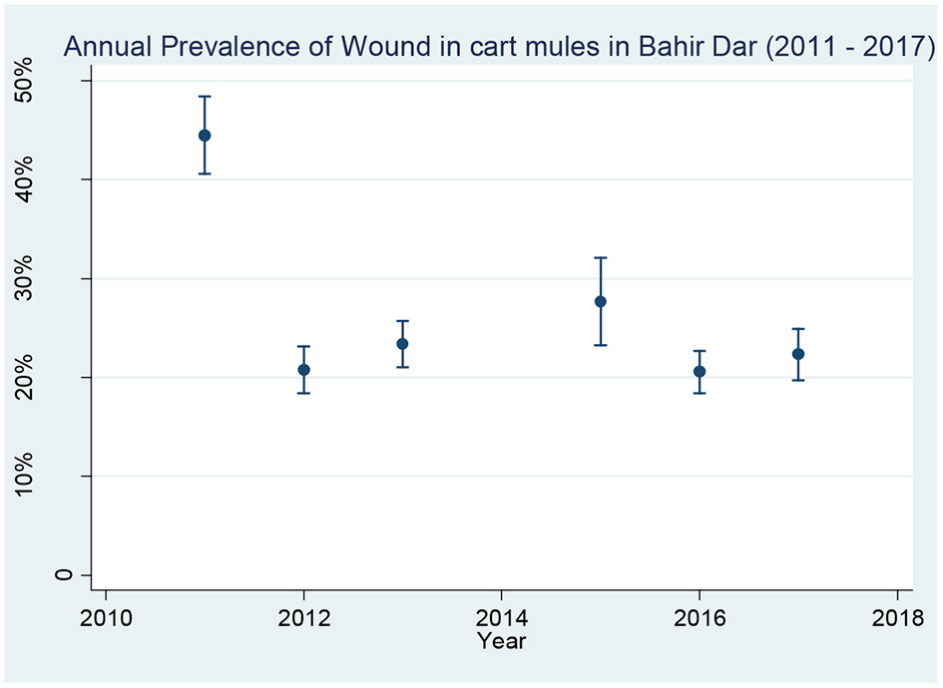
Annual prevalence of open wounds in cart mules in Bahir Dar from 2010 to 2017 [NB: Error bars represent 95% confidence interval around wound prevalence value].

As shown in Table 4, the prevalence of EZL reduced from 23.9 to 5.9% during the course of the project. The prevalence of wounds reduced from 44.3 to 22.2%. The greatest changes came in the first 2 years of the project, with the prevalence of EZL reducing faster than that of wounds. After year 3, the improvements plateaued, with rises in 2015 for wounds and in 2016 for EZL as the project tested handing more responsibility to private veterinary clinics. Despite this, trend analysis showed that the prevalence reductions in both EZL and wounds over the whole duration of the project were statistically significant (χ^2^ = 26.57; *p* < 0.001). Results from the baseline survey in 2010 and the subsequent annual surveys for the prevalence of EZL and wounds during the intervention period from 2011 to 2017 are presented in Figures 3, 4. No data were available for 2014 for reasons external to the project.

The prevalence reduction difference from 2010 to 2017 was 18.0% for EZL and 22.1% for wounds. The prevalence reduction trend analysis for both EZL and wound was statistically significant (χ^2^ = 39.02; *p* < 0.001). Wound data for 2010 were excluded because open wounds and healed wounds had not been put into different categories. An observation during the annual surveys was that while almost all the open wounds continued to originate from poor harnessing, the severity of wounds was reducing with fewer extensive or infected wounds or discharging abscesses, compared to the baseline stage.

#### Stakeholder Contributions

##### Key Informant Interviews

Individual key informant interviews gave insight about how informants understood EZL and related matters, with agreement about the following general themes.

All key informants believed that mules arrive from external markets free of EZL and only become infected in Bahir Dar. Informants agreed that EZL has a devastating socioeconomic effect, and follow-up discussions explored the cost of the mule (~ETB 10,000 = ~GBP 165) compared to the cost of treatment (~ETB 1,000 for early cases). Besides its socioeconomic impact on cart mule business, abandoned mules were a public concern contributing to a poor image of the city. Traffic police complained that abandoned EZL cases were sources of car accidents at various occasions, and an abandoned mule could be a cause of more than one accident over time, particularly at night. Waste collectors believed that terminally sick mules could easily be transported to disposal sites and killed there as it was tiresome to deal with a dead body elsewhere in the city. They suggested for a coordinated effort of stakeholders. Animal health professionals complained about the lack of treatment options and required updated skill sets in all equine practice, not just EZL as a single disease entity. Cart mule association delegates explained how EZL is a real worry to their business. They complained that new owners are a risk because they do not know how to identify, manage, and prevent EZL. They requested that regulatory authorities formalize and develop the sector. There was general agreement that the predisposing factors were wounds mainly from ill-fitting harnessing, poor hygiene, and lack of segregation practices; the main challenges for control are lack of an agreed-upon sound treatment protocol, poor harnessing practice, lack of awareness, lack of mule movement control, and the business being informal and owned mainly by a resource-poor class of community and illiterate people; and EZL could be prevented easily through collaboration of relevant sectors including prevention of wounds, segregation of cases, and removal of abandoned EZL mule. Transport officers believed that in the long term, EZL will not be a problem in Bahir Dar because the cart mule work will soon be replaced by motorized vehicles.

##### FGDs

The results from the individual exercises used in the FGDs are presented below.

*Socio-economic impacts of EZL (brainstorming and discussion*). Muleteers compared the impact of EZL with the impact of other key endemic diseases that affect their cart mules, specifically colic, African horse sickness, and EZL. They described the negative socioeconomic impact of each on the business: colic occurs rarely and kills only one animal at a time, and it can be prevented; African horse sickness is a risk to other mules but comes once in several years and yet kills some mules while other mules recover; EZL is also a risk to other mules, occurs throughout the year, and has no reliable treatment option, and most EZL cases eventually die.

*Predisposing Factors to EZL (Brainstorming, Card Ranking, and Pairwise Ranking)*. Muleteers identified the main predisposing factors for EZL as open wounds, proximity to another mule affected by EZL, owners' lack of knowledge and experience to prevent EZL, fly season, poor harnessing practice, hoof care, and working conditions.

These factors were then debated in more detail using pairwise ranking in which each factor is compared individually to all the others in turn. A summary of the result is presented in Table 5.

**Table 5:** Pairwise ranking of the predisposing factors identified by experienced muleteers during focused group discussions.

**Predisposing factors of EZL**	**Proximity to EZL case (PE)**	**Owner's lack of experience (OE)**	**Fly season (FS)**	**Open wound (OW)**	**Poor harness (PH)**	**Poor hoof care (HC)**	**Working condition (WC)**
**Proximity to EZL case (PE)*^a^***		**PE**	**PE**	**OW**	**PE**	**PE**	**PE**
**Owner's lack of experience (OE)**			OE	OW	OE	OE	OE
**Fly season (FS)**				OW	FS	FS	FS
**Open wound (OW)**					OW	OW	OW
**Poor harness (PH)**						PH	PH
**Poor hoof care (HC)**							HC
**Working condition (WC)*^b^***							
**Total**	OW	PE	OE	FS	PH	HC	WC
**Rank (score^c^)**	1st (6)	2nd (5)	3rd (4)	3rd (4)	4th (2)	5th (1)	6th (0)

a *Proximity at work, feeding, water, grazing, and housing*.

b *Types of work engaged such as transporting construction, logs, and flour*.

c *Score = number of other factors considered more important than during the individual pairwise comparisons*.

Participating muleteers ranked open wounds as the most important factor affecting spread of EZL, followed by proximity to other mules affected by EZL; next were owner's lack of experience and fly season, which were of equal importance; then poor harness was more important than poor hoof care practice; and work type was the least important factor.

*Seasonal Calendar*. The owners concluded that mules get the disease throughout the year; however, new cases were observed more after the long rainy season during the months of September and October, remain low during the dry seasons, and rise again following the small rainy season in April and May of the year.

**Figure 5:**
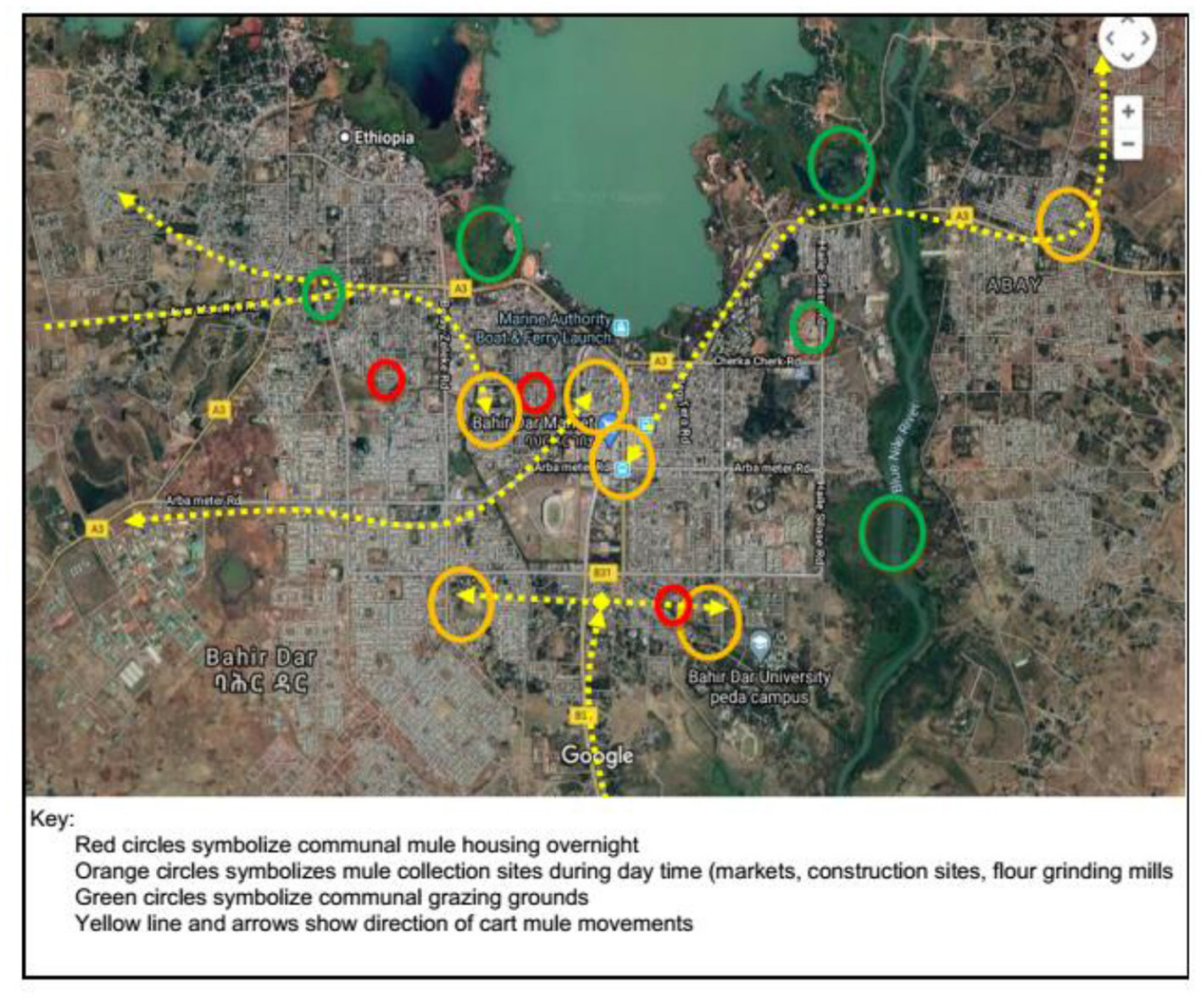
Cart mule movement dynamics and gathering sites in Bahir Dar.

*Participatory Mapping*. Key informants identified cart mule entry points to Bahir Dar, as well as movement and distribution. They located mule pathways and sites where cart mules come together such as markets, construction sites, flour mills, water points, grazing fields, healthcare units, and large group housing sites. The owners worked together to map these using referring points, which were translated onto a Google Map (see Figure 5).

The key informants summarized that cart mules were free to work anywhere in the city, be it markets, construction, or other working sites. There was no restriction of movement for an EZL case until it is abandoned. An abandoned EZL case still had access to socialize with apparently normal mules at feeding and water points, other collection sites, or grazing areas. Abandoned cases stay alive for months, and that is when they remain the source of infection for other mules.

### Results of Implementation

#### Education of Muleteers

Table 6 shows the numbers of muleteers who attended structured, subject-based workshops which aimed to raise animal welfare awareness and change behaviors. The subject areas were mule behavior, handling, and care; mule harnessing practice and road traffic rules; wound management, including application of tincture of iodine; and EZL, i.e., detection and reporting, prevention measures, and euthanasia.

**Table 6:** Numbers of muleteers attending different training sessions.

**Subject/Year**	**Mule behavior, handling, and care**	**Mule harnessing practice; road traffic rules**	**Wound management, including application of tincture of iodine**	**EZL: detection and reporting, prevention measures, and euthanasia**
2011	870	300	169	370
2012	627	624	124	227
2013	580	552	116	380
2014	600	178	73	410
2015	420	164	107	320
2016	280	388	45	187

Examples of how education activities improved the mule-health-related practices of muleteers included increased number of equine cases visiting the public vet clinic; adoption of project EZL treatment protocols with wound management using application of tincture of iodine and gradual acceptance of euthanasia; segregation of infected and non-affected mules at collection and grazing sites; disinfection of harness materials; greater vigilance in reporting suspected cases of EZL as a notifiable disease; and increased use of cart and animal fitted reflectors when driving at night. In general, the muleteers understood that the success and benefit of the intervention relied on shared responsibility, with each taking responsibility not only for their own mule but also for the herd.

Competence levels of individual muleteers are not reported because individual follow-up was difficult, and competence assessment was therefore based on feedback at meetings, field observations at working sites, and observation of changes in practice. Based on these observations, the majority of muleteers were in the “becoming independent” and “independent” category, apart from a few exceptional muleteers who took it upon themselves to mentor others.

Owners with EZL cases were more immediately attentive and engaged than those who had apparently EZL-free mules, but as the intervention progressed, some owners with advanced EZL cases became reluctant to attend the training or treatment clinics because they feared pressure for euthanasia.

The results of the awareness-raising training sessions with traffic police, transport officers, waste collectors, and other municipality development agents came through their changing, more constructive relationships with the muleteers.

Other events, such as World Animal Day, which was observed every year during the first week of October and promoted animal welfare more widely, demonstrated the growth in confidence of project stakeholders who were able to present themselves in a positive light and to show themselves united.

#### Training of Harness Makers

Of the 12 harness makers, eight successfully completed the training and were producing improved prototypes of saddle and humane bits and straps. During the intervention period, the harness makers produced 584 improved cart saddles, 430 humane bits, and 893 canvas straps and collars, which were exchanged with poor traditional types.

#### Training of Animal Health Professionals

Attendance at training sessions for local animal health professionals is presented in Table 7. The training was intensive in terms of time and tasks. Mule behavior training was particularly challenging for trainees, most of whom had no experience in working professionally with mules. For various reasons, some veterinarians were irregular in attendance. Most veterinarians demanded incentives for the follow-up treatments. The owners' pressure to get his mule healed and TDS commitment to invest in the treatment were boosters for the success of the training and treatment. Despite the challenges mentioned above, all clinic veterinary staff achieved independent competence during the course of the project, assessed through long-term clinic follow-up. Two veterinarians from Bahir Dar clinic and two from animal health posts excelled in their competence as trainees and then trainers and played an important role in ongoing project success.

**Table 7:** Trainings provided for animal healthcare professionals and number of attendees.

**Subject/year**	**Mule behavior, handling, and care**	**EZL diagnosis, treatment, and euthanasia**	**EZL epidemiology and principles of herd health**	**Community facilitation skills and equine husbandry**
2011	24	24	24	18
2012	21	18	18	20
2013	21	13	15	18
2014	16	16	14	16
2015	6	6 (4 ToT*^a^*)	8 (4 ToT*^a^*)	
2016			11	11

a *ToT—trainers' training was provided at a later stage so that new staff can be trained by the local veterinarians*.

#### Treatment of Early Cases and Euthanasia of Advanced Cases

Treatment and euthanasia were critical components of the project to remove cases from the herd. For the euthanasia protocol, please see the Supplementary Materials section. Before the project, euthanasia was rarely done and had not been considered as a disease control practice in the area. Constraints were lack of drugs and a system for the disposal of carcasses as well as difficulty to get owners' consent without any compensation.

In piloting this model, TDS made drugs available, trained professionals, and raised public awareness in the perceptions and value of euthanasia. The Bahir Dar City municipality cooperated in the provision of trailers, disposal sites, and organizing associations working on city sanitation for disposal of euthanized mules. Getting owners' consent to euthanize mules with poor EZL prognosis was the inherent challenge throughout the intervention. Nevertheless, a total of 123 mules were euthanized during the period of the project.

Development of cart mule association bylaws helped reinforce good practice among members and influenced non-members through peer association. The Bahir Dar municipality also, for the first time, developed a range of bylaws relating to working equids; however, this came toward the end of the project period, and no mechanism for effective enforcement was developed before the project ended.

### Evaluation of the Project by Signatories

The project was originally signed for 5 years with the intention of piloting a community-based system to control and prevent EZL. The Amhara Regional Livestock Agency, the Bahir Dar City Administration, and Regional Bureau of Transport, all regulated and coordinated by the NGO desk of the Regional Bureau of Finance and Economics, jointly conducted midterm and final evaluations.

The midterm evaluation focused on stakeholder commitment, coordination mechanisms, and testing the impact on the prevalence of EZL. Finding a dramatic reduction in the prevalence of EZL opened the eyes of many stakeholders, particularly the muleteers, animal health professionals, and traffic police.

The final evaluation focused greater emphasis on drawing conclusions about best practice and sustainability. The evaluating team reflected that the project impact was significant and obvious to the general public in Bahir Dar. The most publicly noticeable change was avoidance of abandoned EZL-affected mules in the middle of roads in Bahir Dar city, a prime tourist destination in Ethiopia. The practice of abandoning a mule at the end of its working life to continue to suffer in pain and die of deprivation of food and water is visibly cruel, is a public worry, and portrays a bad image for the city. All parties witnessed and shared success stories: the livestock agency witnessed the treatment success and training modalities for its animal health professionals; the municipality witnessed and appreciated the value of coordination in transforming livelihoods of muleteers; and the traffic police officers witnessed a persistent reduction of cart-mule-associated road traffic accidents.

A learning point was the time needed to establish a community-based project and engage stakeholders. One 2-year project extension was completed to strengthen the intervention, but for reasons beyond its control, the project then ended.

### Outcome of Project Exit Strategy

The objective of an exit strategy is for the project as an entity to end its input, leaving behind a self-sustaining positive impact.

The benefits of the euthanasia program to mule herd health and the mechanism of operation have been recognized in Bahir Dar. A bylaw was established to sustain the change and control and prevent EZL. The articles contained in the bylaw require notification of an EZL case to the nearest traffic police or public veterinary clinic within 48 h, segregation of an EZL case, euthanasia of a terminally sick EZL case, prevention of wound, and registration of new muleteers as they join the business. The Bahir Dar city administration mainstreamed the disposal mechanism of dead mules alongside solid waste.

The traffic regulations mainstreamed animal-powered transport systems into the Bahir Dar traffic public awareness-raising program to minimalize road traffic accidents. Subjects promoted include use of reflectors; improved harness and mule health and welfare; a minimum muleteer age; and skill and knowledge of driving to prevent road traffic accidents.

Trust has been established between Bahir Dar public veterinary clinic service providers and muleteers for the treatment of early cases and euthanasia of terminally sick cases.

However, the long-term sustainability of the EZL project relies on many factors including further formalization of animal-powered transport into the city/regional development program alongside recognition that it is not about to be replaced by mechanized transport and transformation of animal healthcare delivery to include equine medicine and welfare. Also the low status of the sector, the low level of formal education among muleteers with limited alternative livelihood options, the current informal nature of the business, and the influx of new mules into the city remain challenges to sustainability.

## Discussion

### Overview

The TDS tested a community-based model for the control and prevention of EZL and wounds in cart mules in Bahir Dar as part of a wider donkey and mule welfare program in the Amhara Region of Ethiopia. The focus of the project was identified following a welfare assessment using the ‘Hand' tool. Previous studies in Bahir Dar (13, 53) and across multiple countries (54) reported lameness as the most common problem facing working equids. This assessment identified mule–muleteer–societal relationships, wounds, and EZL as the most serious challenges facing mules in Bahir Dar with lameness, nutrition, and abandonment at end of life as additional problems.

The lack of a reliable treatment or a commercially available vaccine limits options for EZL control and prevention. As a result, EZL poses a threat to mule cart businesses and to mule welfare and is the commonest cause of mule abandonment in Bahir Dar. Mules that can no longer pull carts for whatever reason are left to die of thirst and starvation, are often associated with traffic accidents (18, 27, 31), and present a poor image of the city.

TDS recognized that most working equine welfare problems are a result of poor management practices, lack of an affordable healthcare model, and underlying socioeconomic factors and that an effective and lasting solution to EZL control and prevention required involvement of all the relevant stakeholders, including muleteers, service providers, and policy makers, and the interplay of participatory and classical epidemiology. The project built on lessons from previous unsuccessful attempts to control EZL through treatment-only interventions in Bahir Dar and elsewhere (30, 33). TDS identified EZL as a visible indicator of poor welfare for cart mules in Bahir Dar and therefore also of wider program success.

With the ratification of the OIE Working Equid Welfare Standards, the project saw itself as modeling an approach to how these standards might be implemented and therefore increase interest in the outcomes of the work.

### Impact

With a drop in prevalence of EZL from 23.9% (102/430) in 2010 to 5.9% (58/981) in 2017 and of open wounds from 44.3% in 2011 to 22.2% in 2017, the project suggests that prevalence of wounds and EZL can be significantly reduced in working mules through a community-based intervention as described. These drops in prevalence were associated with improved owner husbandry and handling of mules, improved cart and harness design, more mule-friendly municipal traffic practices, more trusting relationships between mule owners and local veterinary staff, improved equine medical competence among local veterinary staff, treatments provided for EZL, reduced abandonment of sick animals, mule community bylaws relating to EZL control put in place, and a means for animals with advanced incurable EZL to be humanely euthanized.

EZL prevalence in 2010 was within the range reported across Ethiopia in cart horses (16, 55), which varied from 39.1% in Mojo to 21.1% in Nazret, and was comparable to reports from Ejaji and Bako where prevalence was 21% in cart mules (17). Prevalence of wounds in 2010 was also comparable to previous reports in Bahir Dar and the adjacent town of Adet (8, 56), where most wounds were also associated with poor harnessing practice (10). Prevalence of EZL in 2017 in Bahir Dar was below any previously published reports of EZL prevalence in working equids in an EZL endemic area of Ethiopia. This reduction in EZL prevalence over the course of the project was significant. The authors are not aware of any other published reports of projects intended to control and prevent EZL in similar or indeed any other circumstances.

The drop in EZL and wound prevalence took place within the first 2 years and over the first 3 years of the project, respectively. These reduced prevalence rates were maintained in the face of year-on-year rises in the population of working mules. It is possible that part of the reduction in prevalence was due to a dilution effect from newly arrived mules, but this factor would not explain the consistent sustained reduction. Equally, the arrival of new mules occurred alongside an increase in mule use and busier collection points, factors that could facilitate an increased rate of EZL spread. During World War II, it was the collection and mixing of horses that challenged the control of EZL (27). With the annually increasing population, we believe that if it were not for the control imposed by the project, the prevalence could have worsened. The rise in the prevalence of EZL in 2016 after a trial in 2015 to hand treatment responsibility to private veterinarians showed how quickly prevalence can rise and highlighted the lack of an economic model for effective private veterinary services in Bahir Dar.

The move in Ethiopia to delineate public and private roles in veterinary services is still in its infancy and beyond the project's scope, but this holds out hope for the future (57). Future projects might help facilitate herd health veterinary service models, possibly with well-established cart mule associations as the herd, whereby instead of being paid for individual treatments, veterinarians are paid collectively against reduction in incidence and prevalence for all association mules.

Possible factors constraining further reductions in prevalence of EZL include the dynamic and unregulated nature of the cart mule business in Bahir Dar with changing demographics; new muleteers with limited experience of wound management and EZL control; mixing of mules during the course of their work, feeding, watering, and general husbandry' unregulated movement around the city of known EZL cases; treatment factors including the investment in time needed; the financial investment in the mule making it difficult for muleteers to accept euthanasia; and the lack of an enabling regulatory framework. Further demographic research on the mule population would have provided rich data but was not possible within the resources of the project. From a welfare point of view, the failure to reduce wound prevalence below 20% was disturbing; however, there was an observed reduction in wound severity during the annual surveys.

In EZL endemic areas, dealing rapidly with EZL cases which act as potential sources of infection, through treatment of treatable cases or euthanasia of untreatable cases, is vital for effective control and prevention. In the project, TDS supplied medicines, equipment, and euthanasia drugs and used each treatment as an opportunity to build competence of animal health professionals. Treatment outcomes were comparable to previously published reports (30, 31, 33, 58, 59).

### Epidemiology

The application of epidemiology and epidemiological tools served two roles in this project. First was to corroborate existing knowledge, take forward understanding about EZL, and inform the work. Second was to engage stakeholders so that they understood and took ownership of the steps needed to control and prevent the disease. The project used both participatory and classical epidemiology methods.

Although elucidating causal relationships between EZL and wounds or other associated factors was not an objective of the study, it was important to corroborate potential risk factors associated with EZL as mentioned in the literature including open wounds, hygiene, and collection site practice (17, 27, 60). Using participatory epidemiology tools, we found that muleteers already recognized proximity to an EZL case in a population (including abandoned mules), open wounds, poor harnessing, new owners with limited experience, poor hoof care, poor working conditions, and fly season as predisposing factors to EZL (Table 5).

Key informants claimed that mules get EZL throughout the year, with new cases being more common following a rainy season (30). However, the project observed the highest numbers of new EZL cases just before the long rainy season, after which untreatable cases were abandoned and die, and the cycle continues. Possible reasons for this include that during the rainy season, economic activities like construction will be reduced, which affects muleteers' income, affecting their expenditure on the care of mules.

The project was not able to explore potential clustering effects of mules temporarily gathered at a given working station. Mules mix fairly flexibly at daytime collection sites, grazing areas, and communal housing sites (see map in Figure 4); EZL-affected individuals are not quarantined; most mules in Bahir Dar work all day with no shift system; owners with more than one mule are generally renting them out. So clustering was considered unlikely to affect results significantly. Nevertheless, ideally, the project would have explored mule demographics in greater detail epidemiologically, socially, and economically.

### Community-Based Approach and Sustainability

The project provides an example of how NGO-led interventions can test new models for change through better access to targeted funding, skill sets, and resources. However, ownership by the local community is needed to make the change last.

In this project, the community-based approach and participatory methods were essential to success by empowering stakeholders, increasing engagement, ensuring local ownership, and building bridges and common understanding between different stakeholder groups. The results of the project are similar to the successes of other community-based animal healthcare initiatives (35, 36, 61).

Encouraging signs of growing stakeholder ownership included development of bylaws and guidelines and use of media to transmit information. The public launch of the project and yearly follow-up events such as World Animal Day proved beneficial in creating a sense of teamwork among the stakeholders and also worked as awareness-raising and advocacy tools.

The successes of the project were achieved despite working animals having no mention in Ethiopia's federal 5-year Growth and Transformation Plans and there being no animal welfare legislation, poor equine health and welfare practices, and little recognition of the valuable social services provided by mules and muleteers. The achievements in shifting viewpoints, particularly among the regulatory authorities, were therefore significant.

Sustaining project impact may require policy changes such as more flexible approaches to pharmaceuticals and equipment procurement by the veterinary regulatory authorities to allow government clinics to respond better to local healthcare priorities or more effective regulation of public and private veterinary roles to allow development of more effective service models.

The sustainability challenges facing the project are common to all donor veterinary projects, and not unique to community-based projects nor to NGO-led projects. The global campaign to eradicate rinderpest took decades and many different approaches, and the current campaign to eradicate peste des petits ruminants is also a long-term campaign. Empowering local veterinary services and involving communities are common to stories of success.

The project responds to a recently published work by Gizaw et al. (62), which assessed veterinary service delivery in Ethiopia, by demonstrating a model for development of services that can work for marginalized mule–muleteer communities in urban settings. The work by Gizaw et al. and this project highlight the need for a study of appropriate veterinary service design and community facilitation skills within the curricula of veterinary training institutes to ensure animal health and welfare professionals' awareness of the different approaches that can be used to achieve improved health and welfare for animals, particularly in resource-poor or otherwise marginalized communities. While holistic socioeconomic transformation of the mule sector will take time, the project has shown what is possible and can act as a seed for change.

### Cost-Effectiveness and Carbon Benefits

The ~ETB 10,000 (~GBP 165) cost of a mule in Bahir Dar reported by muleteers compared to the ~ETB 1,000 cost of EZL treatment if caught early gives a sense of scale to the recent (2021) estimates by Molla et al. (15), in their study of the economic costs of EZL in cart mules and horses in two urban locations in the Amhara Region, with an average annual animal level loss of ETB 6,587 per cart animal per year averaged out between EZL-affected and EZL-unaffected animals.

Using the above figures with mule numbers in Bahir Dar and reduction in EZL prevalence, together with project costs, allows estimation of the cost-benefit. Figures are not presented for this project because they are broad approximations, but they do suggest that there should be a cost-benefit for repeating this work where there are working mules in EZL endemic areas, that the approach could be institutionalized sustainably within public/private veterinary services in Ethiopia (63, 64), and that as the confidence of muleteers and other stakeholders grew, they might realize the benefits of investing in the improvements, including veterinary treatments. Future projects would do well to include economic analysis into their work from the start through collaboration with socioeconomists, ideally from local institutes.

It is also worth noting that Molla et al. (15) did not specifically include additional potential benefits that might accrue to the project from reduced wounds and increased work efficiency from improved communication between mules, muleteers, and municipality. Nor do they include potential carbon/climate/ecosphere-related benefits.

High-welfare mules, working safely and efficiently, are an effective low carbon form or transport, particularly for short distances (65). Throughout the project, transport officials in Bahir Dar held to the idea that mule transport will be replaced by motorized, currently fossil-fuel powered, vehicles, even in the face of year-on-year increases in mule numbers. Nevertheless, they also, for the first time, introduced municipal bylaws to facilitate more efficient, high-welfare mule transport. This demonstrates that community-based projects might play a part in establishing an environment conducive to low-carbon transportation.

### Study Limitations and Challenges

In this study, the socioeconomic conditions and demographic volatility of working mules made follow-up of individual new cases problematic and unaffordable, particularly as EZL and wounds are generally protracted conditions requiring complex interventions. Elucidation of causal relationships between associated risk factors was not pursued as a project priority. To do this within the constraints of such a project would face ethical challenges.

The challenge of exploring clustering effects has been discussed above alongside the desirability for future projects to explore other epidemiological, social, and economic aspects of the cart mule sector, possibly through using a cross-disciplinary team with involvement from local institutes.

Sampling was challenging because of the dynamic nature of the mule population. The plan was to do a full census every year; however, this was not possible in some years. For sample surveys in 2015 and 2017, to reduce the duration of work disruption for muleteers and to ensure representation, mules were mostly examined at their working stations. At times, this posed challenges to the practicality of our systematic random sampling strategy of sampling every other mule (e.g., in a work station where we found only one mule). While there is a possibility that this might introduce sampling bias, we went beyond calculated sample size to minimize this possibility. The risk that vets drawn toward EZL cases despite the sampling methodology might overestimate EZL prevalence was also a possibility.

Unlike most towns, in Bahir Dar, equine power transport is limited to cart mules (6). With increasing urbanization and a high unemployment rate, rapidly growing cart mule numbers presented a challenge to the project as mostly inexperienced new muleteers with new mules arrived. Nevertheless, established muleteers, trained animal health professionals, and traffic police officers took responsibility to engage with new arrivals, and EZL and wound prevalence reductions remained comparatively low.

While muleteers in Bahir Dar are all male, this is not the case elsewhere in Ethiopia. There appears to be a more even balance of female and male equine owners in rift valley towns near Ziway where EZL is also present. In future projects, it would be useful to explore similarities and differences between male and female muleteers.

While case definitions for the different stages of EZL may seem clear to animal health professionals, all treatment protocols require the compliance of muleteers, each of whose social and economic circumstances are different. Some with advanced case of EZL chose to move to the periphery of the town and work outside normal hours to continue making money from their infected mule in the face of social disapproval. Future projects might want to consider compensation or insurance schemes to improve compliance and help reduce prevalence still further; however, these need to be carefully designed and run, both with stakeholder involvement, if they are to be effective. If the status and economic security of the cart mule sector were to rise, these options might become more feasible and acceptable.

The continuing belief by planners and regulators that cart mules will be replaced by motor transport has held back the development of animal-powered transport despite its social value. The project has helped break down this prejudice slightly, but ideally, animal-powered transport should be included in government development plans. Similarly, a lack of understanding about animal welfare presents a challenge to improving the efficiency and effectiveness of the sector.

### Applicability and Replicability

The project is applicable not only to other cities in Ethiopia with endemic EZL but also to animal healthcare challenges in other circumstances, including other countries with EZL (62). Successful replication requires recognition of the time needed to build the project on a secure foundation of stakeholder engagement from community to regulatory level. This includes establishing a common understanding of the epidemiology prior to the intervention and close engagement with veterinary and transport service regulatory bodies. Specifically for EZL, early development of community-level and municipality bylaws would be helpful and a compensation or insurance mechanism to facilitate euthanasia of advanced cases would be worth exploring.

## Conclusions

The project achieved its aim of demonstrating an affordable sustainable approach to improving mule welfare with a reduction in EZL and wounds in cart mules in Bahir Dar between 2010 and 2017 despite rapidly changing mule demographics.

Every step was manageable within existing local institutions, with locally available resources, and economic considerations suggest it could be affordable. Although, for reasons outside its control, the project could not go far enough in embedding the processes, much of the routine work was already being handed over.

To replicate the intervention in other endemic areas, we recommend engaging with stakeholders and establishing the epidemiology at the start, developing bylaws, and exploring an insurance or compensation mechanism for euthanasia cases.

Participatory methodologies were essential for engaging stakeholders and empowering communities, and the lessons learnt show the value of a community-based approach to infectious disease control alongside wider human and animal welfare benefits, particularly in resource-poor or otherwise marginalized communities.

With the OIE Working Equid Welfare Standards now adopted internationally, the authors suggest that integrated community-based interventions are a useful approach to EZL control and prevention in endemic areas within wider working equid welfare improvement programs.

## Data Availability Statement

The raw data supporting the conclusions of this article will be made available by the authors, without undue reservation.

## Ethics Statement

The project was signed by government authorities following a critical review of the respective experts. For the animal studies, including mule sampling and the overall clinical trial, we obtained ethical approval from Gondar University. Informed consent was also obtained from owners for the participation of their animals in this study. For muleteers and key informants we obtained oral consent from individual participants at each stage of the study. Written informed consent for participation was not required according to the national legislation or the institutional requirements.

## Author Contributions

BD and SB designed and supervised the study. TT, AKassaye, and AKassa implemented the programme on the ground. BD, SB, and AKassaye wrote up the study. All authors contributed to the article and approved the submitted version.

## Funding

This work was supported by The Donkey Sanctuary credited.

## Conflict of Interest

The authors declare that the research was conducted in the absence of any commercial or financial relationships that could be construed as a potential conflict of interest.

## Publisher's Note

All claims expressed in this article are solely those of the authors and do not necessarily represent those of their affiliated organizations, or those of the publisher, the editors and the reviewers. Any product that may be evaluated in this article, or claim that may be made by its manufacturer, is not guaranteed or endorsed by the publisher.

## References

[1] CSA (Central Statistics Agency). Agricultural Sample Survey 2020/21. Statistical Bulletin 589, Vol. 2 Report on Livestock and Livestock Characteristics (Private Peasant Holdings). (2021). Addis Ababa: Central Statistical Agency (CSA).

[2] Behnke R Fitaweke M. The contribution of livestock to the Ethiopian economy - Part II, IGAD LPI Working Paper N° 2–11, Djibouti(2011). p. 20-22. Available online at: https://core.ac.uk/download/pdf/132642443.pdf-

[3] Ochieng F Alemayahu M Smith D. Improving the productivity of donkeys in Ethiopia. In: Responding to the Increasing Global Demand for Animal Products: Programme and Summaries 4, London, DFID (2004). p. 93–94. Available online at: https://assets.publishing.service.gov.uk/media/57a08cb6e5274a31e00013b2/R7350j.pdf

[4] Temesgen Z Sitota T. The welfare issues of working equine in Ethiopia: a review. Europ J Biol Sci. (2019) 11:82–90. Available online at: https://www.idosi.org/ejbs/11(3)19/3.pdf

[5] World Bank 2019. Population total – Ethiopia (1995-2019). Available online at: https://data.worldbank.org/indicator/SP.POP.TOTL?end=2019&locations=ET&start=1995. Retrieved May 29, 2021.

[6] Bekele H Teshome W Nahom W Legesse G. Socioeconomic impact of epizootic lymphangitis in cart mules in Bahir Dar city, North West Ethiopia (Conference presentation). Proceedings of the 7th international colloquium on working equids (July 2014), Royal Holloway, University of London, United Kingdom (2014). Available online at: https://www.researchgate.net/publication/302499290_7th-colloquium-on-working-equids-proceedings

[7] Fentie G Teka F Fikadu A Ayalew N Tsegalem A. Injuries in donkeys and mules: causes, welfare problems and management practices in Amhara Region, Northern Ethiopia. Am-Euras J Sci Res. (2014) 9:98–104. 10.5829/idosi.aejsr.2014.9.4.21802

[8] Dressie D Temesgen W Yenew M. Study On Welfare Assessment of Cart Pulling Mule in Bahir Dar Town, Northwest Ethiopia. Rep Opinion. (2017) 9:73–86. 10.7537/marsroj090717.12

[9] Perry B. We must tie equine welfare to international development. Veterinary Record. (2017) 181:600. 10.1136/vr.j5561 29192048

[10] Meselu D Abebe R Mekibib B. Prevalence of epizootic lymphangitis and bodily distribution of lesions in cart-mules in Bahir Dar town, northwest Ethiopia. J Vet Sci Technol. (2018) 9:1–4. 10.4172/2157-7579.1000509

[11] Solomon T Mussie HM Fanaye S. Assessment of carting equine welfare and management practice in Bahir Dar town. Int J Adv Res Biol Sci. (2016) 3:100–112. Available online at: https://ijarbs.com/pdfcopy/dec2016/ijarbs13.pdf

[12] Mulualem T Mekonnen A Wudu T. Seroprevalence and risk factors of African Horse Sickness in mules and donkeys in selected sites of West Amhara Region, Ethiopia. Afr J Microbiol Res. (2012) 6:4146–51. 10.5897/AJMR11.1475

[13] Ali A Orion S Tesfaye T Zambriski JA. The prevalence of lameness and associated risk factors in cart mules in Bahir Dar, Ethiopia. Trop Anim Health Prod. (2016) 48:1483–9. 10.1007/s11250-016-1121-7 27587009

[14] Takele B Nibret E. Prevalence of gastrointestinal helminthes of donkeys and mules in and around Bahir Dar, Ethiopia. Ethiop Vet J. (2013) 17:13–30. 10.4314/evj.v17i1.2

[15] Molla AM Fentahun T Jemberu WT. Estimating the economic impact and assessing owners' knowledge and practices of epizootic lymphangitis in equine cart animals in Central and South Gondar Zones, Amhara Region, Ethiopia. Front Vet Sci. (2021) 8:673442. 10.3389/fvets.2021.673442 34222399 PMC8245057

[16] Amenil G Siyoum F. Study on histoplasmosis (epizootic lymphangitis) in cart-horses in Ethiopia. J Vet Sci. (2002) 3:135–40. 10.4142/jvs.2002.3.2.135 12441683

[17] Ameni G Terefe W A. cross-sectional study of epizootic lymphangitis in cart-mules in Western Ethiopia. Prev Vet Med. (2004) 66:93–9. 10.1016/j.prevetmed.2004.09.008 15579337

[18] Ameni G. Epidemiology of equine histoplasmosis (epizootic lymphangitis) in carthorses in Ethiopia. Vet J. (2006) 172:160–65. 10.1016/j.tvjl.2005.02.025 16772141

[19] Ameni G. Pathology and clinical manifestation of epizootic lymphangitis in cart mules in Ethiopia. J Equine Sci. (2007) 18:1–4. 10.1294/jes.18.1

[20] Endebu B Roger F. Comparative studies on the occurrence and distribution of Epizootic lymphangitis and Ulcerative lymphangitis in Ethiopia. Int J Appl Res Vet Med. (2003) 1:219–222. Available online at: http://www.jarvm.com/articles/Vol1Iss3/Endebu.htm

[21] Scantlebury CE Zerfu A Pinchbeck GP Reed K Gebreab F Aklilu N. Participatory appraisal of the impact of Epizootic lymphangitis in Ethiopia. Prev Vet Med. (2015) 120:265–76. 10.1016/j.prevetmed.2015.03.012 25980831

[22] Blakeway S. The multi-dimensional donkey in landscapes of donkey-human interaction. Relat Beyond Anthropocentrism. (2014) 2:59–77. 10.7358/rela-2014-001-blak

[23] Cousquer G. Promoting pack mule welfare on expedition. Prof Mountaineer. (2015) 9:14–6. Available online at: https://www.travindy.com/2015/12/pack-mule-welfare-abuses-in-mountain-tourism/

[24] Cousquer G. Knowing the mule: Faring well in Moroccan mountain tourism. (PhD thesis), University of Edinburgh, Edinburgh. (2018).

[25] Blakeway S Cousquer GO. Donkeys and mules and tourism. In N. Carr & D. M. Broom (Eds.), Tourism and animal welfare (2018). p. 126–3. Wallingford: CAB International. 10.1079/9781786391858.0126

[26] Scantlebury C Reed K. Epizootic lymphangitis. In: Infectious Diseases of the Horse, Ed: TS Mair & RE Hutchinson, Equine Veterinary Journal Ltd, Ely, Cambridge shire, United Kingdom (2009). p. 397–406.

[27] Al-Ani F. Epizootic lymphangitis in horses: a review of literature. Rev Sci Tech Off Int Epiz. (1999) 18:691–699. 10.20506/rst.18.3.1186 10588013

[28] Taboada J. Epizootic lymphangitis. In: Kahn CM, Line S, Aiello SE, editors. The Merck veterinary manual. Whitehouse Station, NJ: Merck and Co. (2018). Available online at: https://www.msdvetmanual.com/generalizedconditions/fungal-infections/epizootic-lymphangitis. (accessed 29 May 2021).

[29] Carter GR Chengappa MM Claus W Rikihisa Y. Essentials of Veterinary Bacteriology and Mycology. (4th ed.). Philadelphia : Lea & Febiger (1991). p. 284.

[30] Getachew A. Clinical trial of iodides combined with ancillary treatment on Epizootic lymphangitis in cart horses at Debre Zeit and Akaki towns. Faculty Vet Med. (2004)

[31] Mekonnen N Makonnen E Aklilu N Ameni G. Evaluation of berries of Phytolacca dodecandra for growth inhibition of Histoplasma capsulatum var. farciminosum and treatment of cases of epizootic lymphangitis in Ethiopia. Asian Pacific J Trop Biomed. (2012) 2:505–510. 10.1016/S2221-1691(12)60086-0 23569960 PMC3609337

[32] Jones K. Epizootic lymphangitis: the impact on subsistence economies and animal welfare. Vet J. (2006) 172:402–4. 10.1016/j.tvjl.2006.06.003 16899380

[33] Tagesu A. Review on equine Epizootic lymphangitis and its impact in Ethiopia. J Vet Med Res. (2017) 4:1–8. Available online at: https://www.jscimedcentral.com/VeterinaryMedicine/veterinarymedicine-4-1087.pdf

[34] Gardiner A. Veterinary anthropology explored. Vet Rec. (2016) 178:575–6. 10.1136/vr.i2888 27256260

[35] Catley A Blakeway S Leyland T. Community-based animal healthcare: a practical guide to improving primary veterinary services (2002). p. 368.

[36] Catley A Leyland T. Community participation and the delivery of veterinary services in Africa. Prev Vet Med. (2001) 49:95–113. 10.1016/s0167-5877(01)00171-4 11267692

[37] Catley A Alders RG Wood JL. Participatory epidemiology: approaches, methods, experiences. Vet J. (2012) 191:151–60. 10.1016/j.tvjl.2011.03.010 21856195

[38] Alders RG Ali SN Ameri AA Bagnol B Cooper TL Gozali A. Participatory Epidemiology: principles, practice, utility, and lessons learnt. Front Vet Sci. (2020) 7:532763. 10.3389/fvets.2020.532763 33330678 PMC7672004

[39] Mariner J Roeder P. Admassu, B. Community participation and the global eradication of rinderpest. PLA Notes. (2002) 45:29–33. Available online at: https://www.iied.org/pla-45-community-based-animal-healthcare

[40] Mariner JC House JA Mebus CA Sollod AE Chibeu D Jones BA. Rinderpest eradication: appropriate technology and social innovations. Science. (2012) 337:1309–12. 10.1126/science.1223805 22984063

[41] Roeder P Mariner J Kock R. Rinderpest: the veterinary perspective on eradication. Philos Trans R Soc Lond B Biol Sci. (2013) 368:20120139. 10.1098/rstb.2012.0139 23798687 PMC3720037

[42] Samy A Ibrahim MG Mahmod WE Fujii M Eltawil A Daoud W. Statistical assessment of rainfall characteristics in Upper Blue Nile Basin over the period from 1953 to 2014. Water. (2019) 11:468. 10.3390/w11030468

[43] Hart T, Burgess, R,. Beukes O Hart C.. (2005). Reducing pitfalls in agricultural development projects: a case for the Participatory Project Management Cycle (PPMC). S Afr J Agric Ext. (2005) 34:104–21. Accessed online at: https://www.ajol.info/index.php/sajae/article/view/3681

[44] Hart T, Burgess, R,. Hart C. A participatory project management cycle: can it add value to agricultural development? S Afr J Agric Ext. (2005) 34:201–20. Available online at: https://www.ajol.info/index.php/sajae/article/view/3670

[45] Brouwer JH Hiemstra W Martin P. Using stakeholder and power analysis and BCPs in multi-stakeholder processes. PLA Notes. (2012) 65:184–92. Available online at: https://pubs.iied.org/g03412

[46] Michael T Matthew T Wilbur L. A systematic approach to estimating the age of a horse. In: AAEP – Proceedings, (1999). p. 45. Available online at: https://www.dentalage.co.uk/wp-content/uploads/2014/09/martin_mt_1993_daa_horses.pdf

[47] The Donkey Sanctuary. Body Scoring. In: The Clinical Companion of the Donkey. 1st Edition, Linda Evans and Michael Crane (eds), The Donkey Sanctuary, UK (2018). p. 258.

[48] Fowler FJ. Survey research methods. 4th ed. Thousand Oaks, CA: SAGE. (2009). 10.4135/9781452230184

[49] Thrusfield M Christley R Brown H Diggle P French N Howe K. Veterinary Epidemiology, 4th Ed. (2018). John Wiley & Sons Ltd1. 10.1002/9781118280249

[50] IIED 1994. RRA Notes 20: Livestock. Available online at: https://www.iied.org/rra-notes-20-livestock

[51] IIED 2013. PLA Notes. Available online at: https://www.iied.org/participatory-learning-action-pla

[52] ODI. Planning tools: Problem Tree Analysis (2009). Available online at: https://odi.org/en/publications/planning-tools-problem-tree-analysis/. (accessed June 25, 2021)

[53] Meseret B Mersha C Tewodros T Anteneh K Bekele M Nahom W. Lameness and associated risk factors in cart mules in Northwestern Ethiopia. Global Veterinaria. (2014) 12:869–77. Available online at: https://www.idosi.org/gv/gv12(6)14/21.pdf

[54] Pritchard JC Lindberg AC Main DC Whay HR. Assessment of the welfare of working horses, mules and donkeys, using health and behaviour parameters. Prev Vet Med. (2005) 69:265–83. 10.1016/j.prevetmed.2005.02.002 15907574

[55] Hadush B Michaelay M Menghistu H Abebe N Genzebu AT Bitsue HK et al. al. Epidemiology of epizootic lymphangitis of carthorses in northern Ethiopia using conventional diagnostic methods and nested polymerase chain reaction. BMC Vet Res. (2020) 16:375. 10.1186/s12917-020-02582-2 33028302 PMC7541241

[56] Seyoum A Birhan G Tesfaye T. (2015). Prevalence of work related wound and associated risk factors in cart mules of Adet town, North-Western Ethiopia. Am-Euras J Sci Res. (2015) 10:264–71.

[57] Gizaw S Berhanu D. Public-private partnerships (PPPs) for veterinary service delivery in Ethiopia (2019). Nairobi, Kenya: ILRI. Available online at: https://www.ilri.org/publications/public-private-partnerships-ppps-veterinary-service-delivery-ethiopia. (accessed July 7, 2021).

[58] Worku T Wagaw N Hailu B. Epizootic lymphangitis in cart mules: a community-based clinical trial in Bahir Dar, north-west Ethiopia. In: The 6th International Colloquium on Working Equids: Learning from Others. London: The Brooke (2010). p. 256–61.

[59] Hadush B Ameni G Medhin G. Equine histoplasmosis: treatment trial in cart horses in Central Ethiopia. Trop Anim Health Prod. (2008) 40:407–11. 18575967 10.1007/s11250-007-9099-9

[60] Radostits OM Gay CC Hinchcliff KW Constable PD. Veterinary Medicine: A Textbook of the Diseases of Cattle. Horses, Sheep, Pigs and Goats (10th Edition). London, Elsevier Saunders. (2007).

[61] Waziri MI Yunusa KB. The concept and methods of community participation in animal and human disease surveillance. Res Square. (2020). 10.21203/rs.3.rs-22448/v1

[62] Gizaw S Woldehanna M Anteneh H Ayledo G Awol F Gebreyohannes G. Animal health service delivery in crop-livestock and pastoral systems in Ethiopia. Front Vet Sci. (2021) 8:601878. 10.3389/fvets.2021.601878 34164445 PMC8215337

[63] Leonard D. (ed.) Africa's Changing Markets for Health and Veterinary Services: The New Institutional Issues. Macmillan Press, London; St. Martin's Press, New York. (2000)

[64] Leonard D. Structural Reform of the Veterinary Profession in Africa and the New Institutional Economics

[65] Valette D. Invisible Workers: The Economic Contributions of Working Donkeys, Horses and Mules to Livelihoods. The Brooke (2015). Available online at: https://www.thebrooke.org/sites/default/files/Advocacy%20and%20policy/Invisible-workers-report.pdf

