# Supplementary material for: Control and Prevention of Epizootic Lymphangitis in Mules: An Integrated Community-Based Intervention, Bahir Dar, Ethiopia
Source: Front Vet Sci. 2021 Nov 12;8:648267. doi: 10.3389/fvets.2021.648267 (PMC8632952; doi:10.3389/fvets.2021.648267)
Supplement: Supplementary file 1 [file Data_Sheet_1.pdf]

Supplementary Materials:
Control and Prevention of Epizootic Lymphangitis in Mules: An Integrated Community-Based Intervention, Bahir Dar, Ethiopia
Duguma BE, Tesfaye T, Kassaye A, Kassa A and Blakeway S (2021)
Front. Vet. Sci. 8:648267. doi: 10.3389/fvets.2021.648267

Clinical trial of Epizootic lymphangitis (EZL) with treatment protocol

Objectives:
The objective of this clinical trial was to remove as many cases as possible from the rest of the mule population through treatment of early cases and euthanasia of terminal cases. Also, to raise awareness among muleteers, who have previously depended on practices such as cauterization, of the effectiveness of modern treatments at least for early cases, and of ending the suffering of terminal cases. In doing so we aimed to build bridges between local veterinary services and muleteers. It also created a platform to educate the benefits of euthanasia in EZL control and prevention protocol.

Protocol

The protocol was adapted from Getachew Assefa, 1994, DVM Thesis, Addis Ababa University.
For the purpose of this intervention, EZL cases were categorized into 4 clinical stages.

a) Classification of cases and case definition

Table 1: Case definitions of EZL cases

	Cases	Definition
1	Early cases:	An EZL case, with palpable skin nodules along the lymphatic vessels or ulcerating lesions (continuously oozing serous fluid) on the limbs or necks, rarely other body parts; often due to chaffing at the fetlock or harness wounds. One or two parts of the body may be affected.
2	Established case	An established EZL case, increased number of granulomatous nodules with lesions progressed and aligned along the affected limb, neck or head region; lesions might have joined to form a lacerated wound; one or two limbs may be involved; cording of lymphatic becomes prominent only upon palpation and painful; swelling of the affected limb compared to the collateral limb; might have advanced to involve a regional lymph node.
3	Advanced cases (often with guarded treatment outcome)	A chronic EZL case; nodules and ulcerations beyond count; lesions might have joined to form a bigger wound, often involve lower body parts such as inguinal or auxiliary area; cording of lymphatic is common; the swelling or pain might have interfered with the locomotor, visual or respiratory function of the animal. The animal is still working.
4	Terminal stage	Obviously advanced chronic EZL case; characterized by functional impairment of often respiratory, visual or locomotor. Such cases rarely work and usually abandoned. However, they continue to live grazing and scavenging in the urban and per-urban areas, often preferring to walk alone in the middle of tarmac roads to keep flies away. This category is for euthanasia hoping to remove from the habitat with the consent of owners.

The classification and case definition were made clear to all stakeholders through a brochure with pictures and notes, to differentiate in particular between early cases, and advanced and terminal cases. The case definition of the four stages is presented in Table 1.

b) Treatment protocol for Epizootic lymphangitis cases

The treatment protocol is adopted from previous works of Getachew Assefa (6). Parenteral iodides with a simultaneous local application of tincture of iodine, supported with surgical incisions of nodules where required and subsequent dressing.

Excluding advanced and terminal cases was a challenge because of ethical reasons and euthanasia was subjected to owner's consent. Owners were consulted about the probable prognosis of their respective cases and requested to consent to take part.

Supplementary measures that involve owners and other stakeholders included deworming, proper feeding, resting the animal (otherwise advice to reduce the work burden), greater attention by owner to wound prevention and care, and improve harness.

i. Treatment protocol for early and moderate EZL cases

Potassium iodide (KI) was orally administered with soaked wheat bran, and in situations where the mule could not eat, it was administered mixed with water through a nasogastric tube. KI was administered at a dose of 0.1gm/kg body weight of mule once daily. The parenteral dose regimen was continued for the first five days and then every other day for a fortnight and then interrupted for a week to assess for any new nodules appearing on any body part of the animal or healing sign of older lesions.

If there were no new signs of progress in infection, oral administration was stopped and only iodine tincture application continues on lesions that are yet scuffing. Otherwise, if scarification already started, everything stops. EZL lesions do not necessarily heal with a natural wound management cycle, it often fails forming scab instead of scar, indicating presence of active infection underneath. It is only after such infection cycle is over that a firm scar will finally be formed. However, if there are new signs of infection characterized by new nodules in new adjacent areas following the lymphatic or any other part of the body, the same cycle another regimen of parental iodides therapy continues. It will be paused for a week once again to assess the progress of the therapy. Most of the cases under this category heal at this stage, otherwise it proves to be advanced with guarded prognosis.

ii. Treatment Protocol for Advanced EZL cases

All protocols remain similar except that parental iodides need to be administered for the first 10 consecutive days and continued every other day for a fortnight. Number of nodules and ulcers, size of lesions, cording regions, involved lymph nodes, and functional abnormality due to EZL (such as lameness, dyspnea, and ocular lesions) needs to be recorded in detail to monitor the progress impact of the therapy. Cases with lymphadenopathies of the inguinal or auxiliary or peritoneal region involvement need to be explored further, and where necessary surgical aspiration needs to be applied to lymph nodes to discharge the pus, flush with iodine tincture to disrupt the infection.

Monitoring and evaluation of the treatment outcome

Progress of the treatment was monitored with an individual case record that included a through individual case assessment and agreed treatment protocol including duration of treatment; date of onset of treatment; weekly progress assessment including number of nodules, size of ulcerating wound, number of newly emerging nodule (s) along the lymphatic vessels or new body site; diameter of wound or swelling of limbs; as well as number of healing scars. Samples were taken for newly emerging nodules. Recovery of intact yeast cells was an indication of progress of an active infection while disintegrated yeast cells (see Fig. 2 in the body of the main paper) was sign of progress of effective treatment.

Results: Treatment outcomes of EZL cases

Treatment outcomes were classified and defined as cured; guarded, advanced and not cured. A mule case under treatment was defined cured if it cured with the pre-scheduled scheme; guarded if the case did not heal with the pre-scheduled time, and with no progress of infection; advanced if there were signs of new infections and not cured when new infections steadily progress and older sites show sign of relapse. The outcome is summarized in Table 2.

Table 2: Treatment outcomes of EZL cases by year 2011-2016

No of cases /Year	Total Number	Early case (# cured)	Moderate cases (# cured)	Advanced cases (# cured)	Euthanized	Number with No final report
2011	169	42 (24)	101 (41)	26(2)	26	76
2012	124	43 (32)	65(28)	16(3)	17	44
2013	116	51 (41)	53 (21)	12(2)	18	34
2014	73	29(24)	42 (21)	3(0)	16	11
2015	107	43(31)	57(23)	7(2)	34	17
2016	45	27(19)	16 (6)	2(0)	12	8
Total	634	235 (171)	333 (141)	66 (9)	123	190
% Cured		73%	42%	14%		

Source: Summarized from monthly veterinary treatment reports of the project (2011-2016)

Discussion

For understandable reasons, it took time to convince muleteers that the project could reverse cases with modern therapy as they were attached to the traditional therapy of cauterization. At the pilot stage it was common to see owners still applying cauterization on top of the veterinary treatment. Once convinced, the next challenge was defining a cut-off point between treatable and untreatable cases. This required trust as well as ethical, livelihood, and cost-benefit analyses. The intervention and treatment outcomes convinced muleteers of the value of early diagnosis and case classification: earlier cases responded but not advanced

terminal cases. Acceptance of euthanasia for advanced terminal cases took time. Some never accepted it, migrating to work in the periphery of the town.

Some early cases appeared to heal spontaneously within the first few days of therapy and owners would then not complete the course. Some owners with advanced cases and a poor prognosis discontinued treatment for fear of euthanasia.

One lesson was the creation of a common resource center for all cases across all clinics, all following the same protocol, to help manage a municipality-wide prevention programme.
